# miR393 contributes to the embryogenic transition induced in vitro in Arabidopsis via the modification of the tissue sensitivity to auxin treatment

**DOI:** 10.1007/s00425-016-2505-7

**Published:** 2016-04-04

**Authors:** Anna M. Wójcik, Małgorzata D. Gaj

**Affiliations:** Department of Genetics, University of Silesia, Jagiellońska 28, 40-032 Katowice, Poland

**Keywords:** Auxin receptors, *AUXIN F*-*BOX PROTEIN (AFB)*, miRNA, *MIRNA*, Somatic embryogenesis, *TRANSPORT INHIBITOR1* (*TIR1*)

## Abstract

**miR393 was found to control embryogenic transition in somatic cells in Arabidopsis via control of the*TIR1*and*AFB2*auxin receptors genes of the F-box family.**

miR393 molecules are believed to regulate the expression of the auxin receptors of the TAAR clade. Considering the central role of auxin in the induction of somatic embryogenesis (SE) in plant explants cultured in vitro, the involvement of miR393 in the embryogenic transition of somatic cells has been hypothesised. To verify this assumption, the reporter, overexpressor and mutant lines in genes encoded *MIR393* and TIR1/AFB proteins of the F-box family were analysed during SE in Arabidopsis. Expression profiling of *MIR393a* and *MIR393b*, mature miR393 and the target genes (*TIR1*, *AFB1*, *AFB2*, *AFB3*) were investigated in explants undergoing SE. In addition, the embryogenic potential of various genotypes with a modified activity of the *MIR393* and *TIR1/AFB* targets was evaluated. The distinct increase in the accumulation of miR393 that was coupled with a notable down-regulation of *TIR1* and *AFB2* targets was observed at the early phase of SE induction. Relevant to this observation, the GUS/GFP monitored expression of *MIR393*, *TIR1* and *AFB2* transcripts was localised in explant tissue undergoing SE induction. The results suggest the miR393-mediated regulation of *TIR1* and *AFB2* during embryogenic transition induced in Arabidopsis and a modification of the explant sensitivity to auxin treatment is proposed as underlying this regulatory pathway.

**Electronic supplementary material:**

The online version of this article (doi:10.1007/s00425-016-2505-7) contains supplementary material, which is available to authorized users.

## Introduction

Non-coding RNAs encoded by *MIRNA* genes, the so-called microRNAs (miRNAs), regulate gene expression by targeting mRNAs for degradation or translational repression (Axtell 2013). Transcription of *MIRNA* genes results in miRNA precursors (pri-miRNA) that are further proceeded to the final mature miRNA molecules through pre-miRNA intermediates (Bartel et al. 2004). The biogenesis of miRNA requires activity of multiple enzyme complexes and among them the RNase-III-like enzyme DICER-LIKE1 (DCL1) was indicated as playing a central role in the biogenesis of most of the miRNAs identified in plants (Reinhart et al. 2002). DCL1 interacts with other double-stranded RNA (dsRNA) binding proteins, HYPONASTIC LEAVES1 (HYL1) and the zinc-finger protein SERRATE (SE), to promote the efficient and accurate biogenesis of miRNA (Cuperus et al. 2010).

Besides DCL1, three other DCL enzymes (DCL2–DCL4) are encoded in the genome of *Arabidopsis thaliana* (Schauer et al. 2013). In contrast to DCL1, which is involved in the biogenesis of *MIRNA*-encoded RNAs, the activity of DCL2-4 is related to the biogenesis of viral short interfering RNA (siRNA) and endogenous siRNA such as retrotransposon siRNA and trans-acting small interfering RNA (Xie et al. 2004). In addition, DCL4 was recently reported to process some of the “young”, not conserved plant miRNA: miR822, miR839 and miR859 (Tsuzuki et al. 2014). DCL3 was revealed to cognate dsRNAs and produce the siRNAs that work in RNA-directed DNA methylation (Chapman and Carrington 2007).

In Arabidopsis, numerous *dcl1* mutants that display a wide range of developmental defects were isolated (Schauer et al. 2002). Mutants of weak *dcl1* alleles, *dcl1*-*7* (*sin1*-*1: short integument1*-*1*) and *dcl1*-*9* (*caf*-*1: carpel factory*-*1*), have pleiotropic phenotypes including small leaves, late flowering and female sterility. In contrast, the null mutants, *dcl1*-*5* (*sus1*-*5: suspensor1*-*5*) and *dcl1*-*6*, are embryonically lethal (Suarez et al. 2015). In contrast to *DCL1*, mutations in other *DCL* genes do not produce defective phenotypes and double and triple mutants in *DCL2*, *DCL3* and *DCL4* were found to develop normally (Bouché et al. 2006).

The critical role of miRNAs in numerous biological and metabolic processes in plants has been documented, including zygotic embryogenesis (ZE) in which miRNAs were documented to be essential for the proper patterning and morphology of the embryos (Willmann et al. 2011; Vashisht and Nodine 2014; Seefried et al. 2014). During ZE in Arabidopsis, miRNAs were found to repress the embryo maturation process early in embryogenesis via the inhibition of the master regulators of ZE, including *LEAFY COTYLEDON 2* (*LEC2*) and *FUSCA3* (Willmann et al. 2011). This documented substantial impact of miRNAs on the control of ZE implicates the involvement of these molecules in the regulation of the somatic counterpart of ZE, i.e. somatic embryogenesis (SE) induced in vitro in cultured explants (Zimmerman 1993). In line with this assumption, a global analysis of the SE-related transcriptomes of *Oryza sativa* (Chen et al. 2011a), hybrid yellow poplar (Li et al. 2012), *Larix laptolerix* (Zhang et al. 2012), *Dimocarpus longan* (Lin and Lai 2013) and *Gossypium hirsutum* (Yang et al. 2013) revealed numerous *MIRNA*/miRNAs with differential expression levels. In addition, stem-loop qRT-PCR revealed differential accumulation of various miRNAs at different stages of SE in *C*. *sinensis* (Wu et al. 2011).

Among the candidate *MIRNAs*/miRNAs with a significant impact on SE induction, those related to auxin responses should be considered due to the central role of auxin signalling/metabolism in embryogenic induction in vitro (Jiménez 2005). In support of this assumption, auxin-related miRNA was identified in the embryogenic cultures of plants and among them miR393 was found in cotton (Wu et al. 2011; Yang et al. 2013). In Arabidopsis, analysis of over 190 *MIRNA* genes showed over twenty genes that were differentially regulated during SE induction and among them *MIR393* genes, *MIR393a* and *MIR393b*, were found to be significantly up-regulated (K. Szyrajew, D. Bielewicz, Z. Szweykowska-Kulińska, A. Jarmłowski and M. D. Gaj, data not published). In Arabidopsis, *MIRNA393* genes encode miR393 of the regulatory functions in the auxin responses that are associated with various developmental processes (Navarro et al. 2006; Si-Ammour et al. 2011). In planta, miR393 were indicated as targeting *TIR1* (*TRANSPORT INHIBITOR1*), *AFB1* (*AUXIN F*-*BOX PROTEIN1*), *AFB2* and *AFB3* (Navarro et al. 2006), which encode members of the TIR1/AFB clade of auxin receptors (TAARs) in the AFB family of plant F-box proteins (Mockaitis and Estelle 2008). Relevant to the essential role of miR393-mediated regulation in ZE of Arabidopsis (Nodine and Bartel 2010), the target mRNAs, *TIR1*, *AFB* and *AUXIN RESPONSE FACTORS* (*ARFs*) were detected as being active in the SE transcriptomes of cotton (Yang et al. 2013), longan (Lin and Lai 2013) and Arabidopsis (Gliwicka et al. 2013; Wickramasuriya and Dunwell 2015).

The aim of the study was to gain more insight into the role of miR393 in the auxin-related mechanisms that operate in SE induction. To this end, we used an embryogenic culture of Arabidopsis, a model plant that offers versatile tools for the functional analysis of genes (Provart et al. 2015). The expression level of *MIR393*, mature miR393 and the target genes (*TIR1* and *AFB*) were evaluated during the course of SE. In addition, the reporter, overexpressor and mutant lines were studied to reveal the relation between the activity of the studied genes and the embryogenic potential of the explants. The distinct increase in the accumulation of miR393 that was coupled with a notable down-regulation of *TIR1* and *AFB2* targets was observed at the early phase of SE induction. The study provides some evidence about the miR393-mediated regulation of embryogenic transition in Arabidopsis and a modification of the explant sensitivity to auxin treatment is assumed to be involved in this regulatory pathway.

## Materials and methods

### Plant material and growth conditions

The *Arabidopsis thaliana* (L.) Heynh. Columbia (Col-0) parental genotype and the transgenic lines *afb2*-*3*, *TIR1pro::EGFP*, *AFB1pro::EGFP*, *AFB2pro::EGFP*, *AFB3pro::EGFP* were supplied by Nottingham Arabidopsis Stock Center (NASC). The T-DNA insertional mutants *dcl1*-*6*, *dcl2*-*1 dcl3*-*1 dcl4*-*2* (hereafter noted *dcl2/3/4*), *miR393a*-*1*, *mir393b*-*1*, double mutant *miR393a*-*1 miR393b*-*1* (hereafter noted *mir393a/miR393b*) and the reporter lines *proMIR393a::GUS* and *proMIR393b::GUS*, were kindly provided by Dr. F. Vazquez (University of Basel, Basel, Switzerland). The *35S::MIR393a*, *35S::MIR393b* and *tir1*-*1* seeds were kindly provided by Prof. Mark Estelle (University of California at San Diego, USA). The characteristics of the transgenic genotypes that were used in the study are presented in Supplementary Table S1.

### In vitro culture of immature zygotic embryos (IZE) explants

#### Somatic embryogenesis induction

Immature zygotic embryos (IZEs) of different genotypes were used as explants to establish an in vitro culture. Due to the embryonic lethality of the *dcl1*-*6* mutation, the IZEs that were homozygous for the mutation were isolated from the segregating *dcl1*-*6/*+ heterozygote. To induce SE, IZEs at the stage of green, fully developed cotyledons were cultured following a standard protocol (Gaj 2001). The standard medium used for SE induction (E5) contained a basal B5 medium (Gamborg et al. 1968) supplemented with 20 g L^−1^ sucrose, agar (8 g L^−1^) and 5 μM of 2,4-D (2,4-dichlorophenoxyacetic acid) (2,4-D, Sigma). In some experiments, 2,4-D at additional concentrations (0.1; 1.0; 10 and 30 μM) was used. As a control culture, an auxin-free (E0) medium was used that resulted in the development of IZEs into seedlings.

#### Induction of organogenesis (ORG)

Shoot organogenesis (ORG), was induced in a culture of IZEs that was cultured for 7 days in a liquid callus induction medium (CIM) with 2.2 µM 2,4-D and 0.2 µM kinetin (Feldman and Marks 1986) and subsequently transferred to a solid shoot induction medium (SIM) supplemented with 0.5 µM naphthalene acetic acid (NAA) and 4.4 µM benzyl adenine (BAP) according to Kraut et al. (2011).

#### Plant growth and in vitro culture conditions

The plants used as the source of the IZE explants were grown in Jiffy-7 pots in a walk-in type green room at 21 ± 1 °C under a 16 h/8 h photoperiod of 100 µM m^−2^s^−1^ white, fluorescent light. The plant material that was grown in sterile conditions, i.e. in vitro cultured explants, were kept in a growth chamber at 21 ± 1 °C under a 16 h/8 h photoperiod of 40 μM m^−2^s^−1^ white, fluorescent light.

#### Evaluation of the culture morphogenic capacity

The explant capacity for SE and ORG was evaluated in 21-day-old cultures and two parameters were calculated—efficiency (the percentage of explants that formed somatic embryos/shoots) and productivity (the average number of somatic embryos/shoots produced per explant). All of the culture combinations were evaluated in three replicates and at least 30 explants (ten explants/Petri dish) were analysed per one replicate.

#### RNA isolation and RT-qPCR analysis

An RNAqueous kit (Ambion) was used to isolate total RNA from the IZE explants induced on an auxin (E5) and auxin-free (E0) medium at 0, 5, 10 and 15 days. Depending on the age of the culture, from 300 (0 day) to 100 (10 days) explants were used for RNA isolation. The concentration and purity of RNA was evaluated with a ND-1000 spectrophotometer (NanoDrop). To control DNA contamination, RNA was treated with RQ1 RNase-free DNase I (Promega) following the manufacturer’s instructions. First strand cDNA was produced in a 20 L^−1^ reaction volume using a RevertAid First Strand cDNA Synthesis Kit (Fermentas). The product of reverse transcription was diluted with water at a 1:4 ratio and 2.5 L^−1^ of this solution was used for RT-qPCR reactions. RT-qPCR was carried out in a 10 L^−1^ reaction volume using a LightCycler^®^ 480 SYBR Green I Master (Roche). The primers that were relevant to the genes being studied were used in the RT-PCR analysis (Supplementary Table S2).

A LightCycler 480 (Roche) real-time detection system was used under the following reaction conditions: denaturation one repeat of 10 min at 95 °C, followed by 45 repeats of 10 s at 95 °C, 8 s at specific for primers temperature, 12 s at 72 °C and 5 s at 80 °C. Denaturation for the melt curve analysis was conducted at 95 °C followed by 15 s at 65 °C and 95 °C (0.1 °C/s for fluorescence measurement).

#### miRNA isolation, stem-loop RT-PCR and RT-qPCR analysis

A mirVana™ Kit was used to isolate miRNAs from the IZE explants induced on an auxin (E5) medium for 0, 5, 10 and 15 days. Depending on the age of the culture, from 2000 (0 day) to 250 (10 days) explants were used for miRNA isolation. The concentration and purity of miRNA was evaluated with a ND-1000 spectrophotometer (NanoDrop). The oligonucleotides design, stem-loop RT and real time qPCR were performed according to Speth and Laubinger (2014). The primers sequences used in the study are listed in Supplementary Table S3.

Primary data analysis was performed using LightCycler Software 4.0 (Roche). Relative RNA levels were calculated and normalised to an internal control—the *At4g27090* gene encoded 60S ribosomal protein (Thellin et al. 1999). In all of the analysed tissue samples, the control gene displayed a constant expression pattern with Cp = 19 ± 1.

The plant tissues for the analysis of gene expression were produced in three biological replicates and two technical replicates of each repetition were carried out. The relative expression level was calculated using $2^{{ - \varDelta \varDelta C_{\text{T}} }}$ where ∆∆*C*_T_ represents ∆*C*_T_^reference condition^ − ∆*C*_T_^compared condition^.

#### Histological analysis

All of the explants derived from the lines with GUS constructs were stained in a standard X-Gluc (5-bromo-4-chloro-3-indolyl β-d-glucuronide cyclohexylammonium salt) (Sigma Aldrich) solution at 37 °C for 12 h as described by Jefferson et al. (1987). The expression of the GUS signal was inspected in the IZEs of the analysed lines using a Zeiss Stemi 2000-C microscope and images were saved as jpg files with an Axi-Vision Camera.

Green fluorescence protein (GFP) fluorescence was excited with a multiband argon laser at 100 mV (Melles Griot BV) and a wavelength of 488 nm. Image processing was performed using the FLUOVIEW computer program (Olympus, version 1.6).

#### Data presentation and statistical analysis

The figures show the averages with the standard deviations. To calculate the significant differences (at *P* = 0.05) between the compared samples, the Student’s *t* test was applied.

## Results

### DCL1 is required for somatic embryogenesis induction

**Fig. 1:**
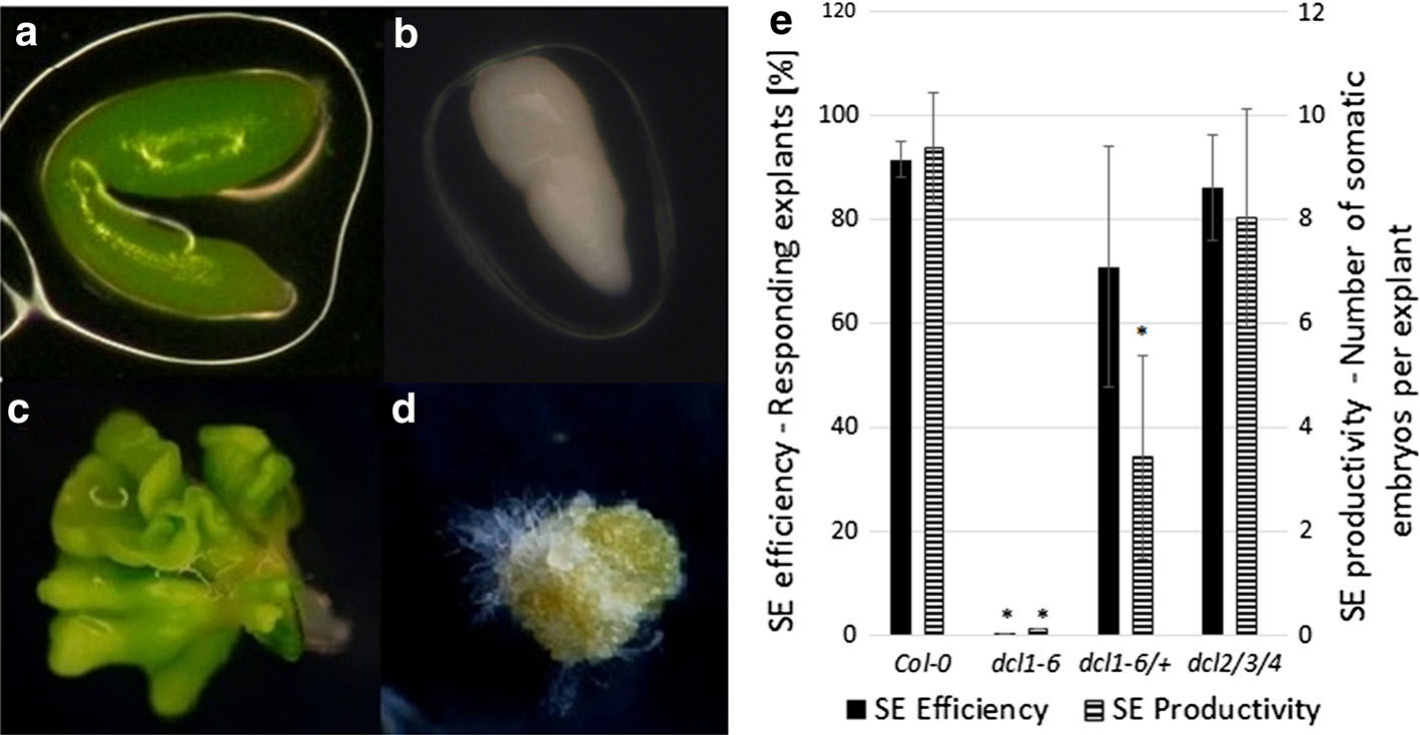
The capacity for SE of *dcl* mutants. Morphology of IZE explants of a highly embryogenic Col-0 genotype (**a**, **c**) and a *dcl1*-*6* mutant that was arrested in embryo development (**b**, **d**) cultured in vitro on an E5 medium for 0 day (**a**, **b**) and 21 days (**c**, **d**). Somatic embryos (**c**) and a non embryogenic callus (**d**) developed from Col-0 and a mutant explant, respectively. SE efficiency and SE productivity (**e**) of Col-0, *dcl1*-*6* and *dcl2/3/4* mutants. *Asterisks* values significantly different from the parental Col-0 genotype (*P* < 0.05; *n* = 3 ± SD)

Two mutants that were impaired in the production of functional DCL proteins were analysed in terms of their embryogenic potential in vitro—*dcl1*-*6* (Schauer et al. 2002) and the triple mutant *dcl2/3/4* (Garcia-Ruiz et al. 2010). It was found that the IZEs that were homozygous for *dcl1*-*6* mutation were arrested early in development (Fig. 1a, b). The embryos cultured on the E5 auxin medium were unable to induce SE and they produced a non-embryogenic callus in contrast to the Col-0 explants that developed somatic embryos efficiently (Fig. 1c, e). A decreased capacity for SE was also observed in the explants isolated from *dcl1*-*6/*+ plants; however, due to the recessive character of *dcl1*-*6* mutation, the embryogenic response of this culture was less impaired. In contrast to *dcl1*-*6*, the *dcl2/3/4* mutant displayed a high embryogenic potential that was comparable to Col-0 culture (Fig. 1e).

These results clearly demonstrated that unlike DCL2, DCL3 and DCL4, the DCL1 function is required for SE induction. Thus, given the key role of this protein in the biogenesis of almost all miRNA, the essential functions of miRNA in embryogenic transition that is induced in vitro in Arabidopsis might be expected.

### *MIR393* genes and their mature products are active in SE

**Fig. 2:**
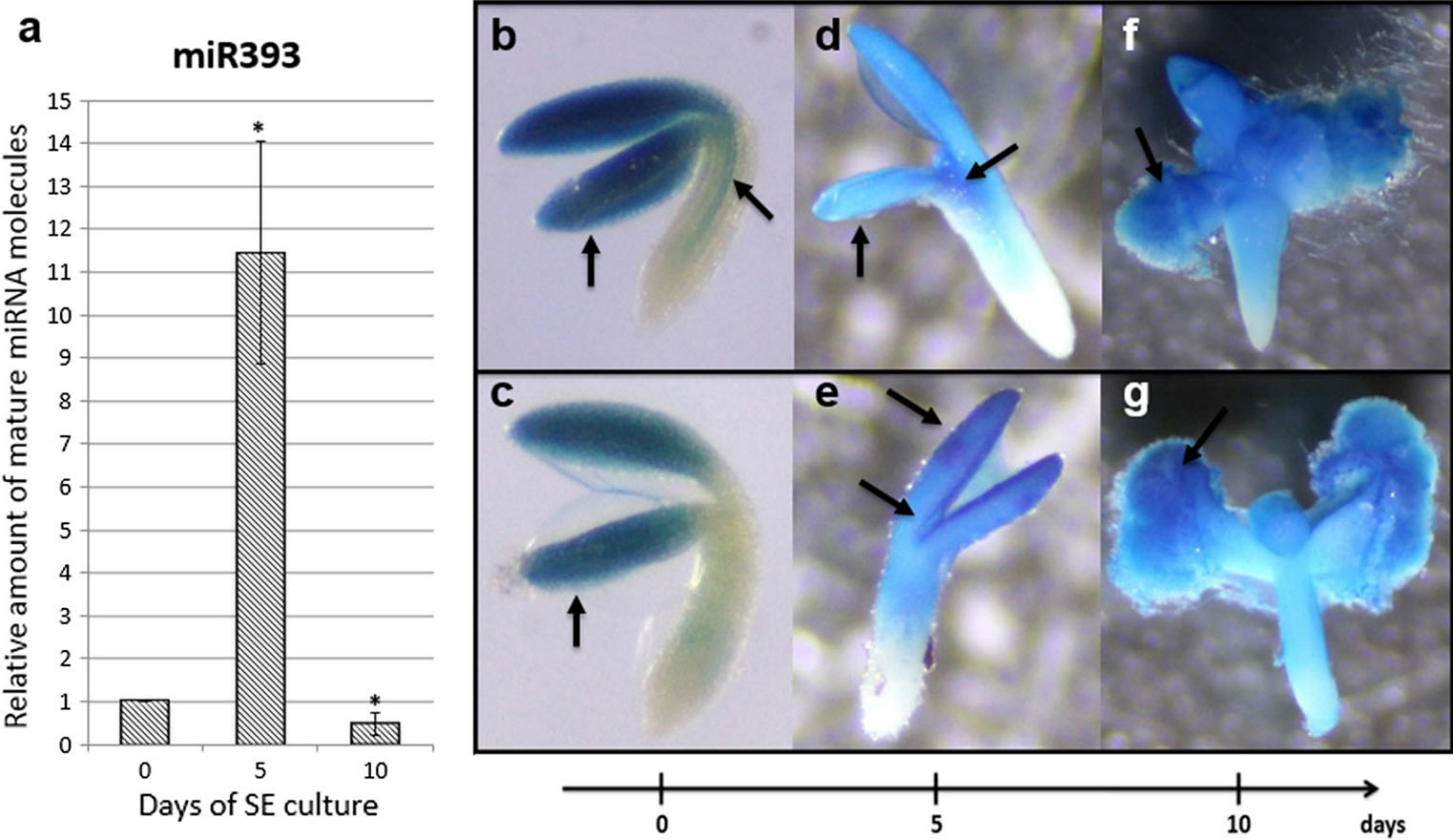
Expression of miRNA/*MIRNA* in embryogenic culture. **a** Relative amount of mature miR393 molecules at 0, 5th and 10th day of Col-0 SE culture. *Bars* represent standard deviation (*n* = 3). Relative transcript level was normalised to the internal control (*At4g27090*) and calibrated to the 0 day of culture. *Asterisks* values significantly different from the 0 day of culture (*P* < 0.05; *n* = 3 ± SD). Expression patterns of GUS-monitored promoters of *MIR393a* (**b**, **d**, **f**) and *MIR393b* (**c**, **e**, **g**) genes at 0 day (**b**, **c**), 5th day (**d**, **e**) and 10th day (**f**, **g**) of SE culture. *Arrows* indicate GUS signal in SE-involved tissue (cotyledons and in the vicinity of SAM)

A survey of *MIRNA* gene transcripts in the embryogenic culture of Col-0 explants indicated a distinct up-regulation of *MIR393a* and *MIR393b* transcripts, which were accumulated up to 53 and 27 fold, respectively (Supplementary Fig. S1). To verify if the activation of *MIR393* expression in the SE-induced explants may result in the production of mature miR393 molecules, stem-loop RT-qPCR analysis was performed. The analysis showed different levels of mature miR393 at all of the monitored time points (0, 5 and 10 days) of the Col-0 embryogenic culture (Fig. 2a). Transient increase in the level of mature miR393 (up to 11-fold) that was observed in the 5 days culture was associated with the early phase of SE induction, what is important for an auxin-related mechanism of SE induction.

A reporter line with promoter::GUS fusions was used to track the spatio-temporal pattern of the expression of *MIR393a* and *MIR393b* genes in the explants that were undergoing SE. The analysis showed that different patterns of GUS-monitored *MIR393a* and *MIR393b* expression were found in freshly isolated explants (0 day). A *MIR393a* GUS signal was observed in the cotyledons and vascular tissue of the hypocotyl part of the IZE (Fig. 2b), while a *MIR393b* signal was only detected in the cotyledons (Fig. 2c). In explants that were induced towards SE and treated with 2,4-D for 5 days, both of the *MIR393* genes were found to be active in an apical part of the IZEs and an especially strong GUS signal was observed in the explant parts that are involved in embryogenic transition, i.e. in the adaxial parts of the cotyledons and in the vicinity of the shoot apical meristem (SAM) (Fig. 2d, e). In the advanced SE culture (10 days), *MIR393* GUS signal was highly accumulated in the cotyledons- and SAM-derived embryogenic tissue developing somatic embryos (Fig. 2f, g).

Collectively, the results of the analyses of the mature miR393 and GUS-reporter lines showed that *MIR393* genes are active in tissue undergoing embryogenic transition and that their mature transcripts are produced, which suggests the engagement of miR393 in the mechanism of SE induction in Arabidopsis.

### Functional analysis of *MIR393* genes in SE

**Fig. 3:**
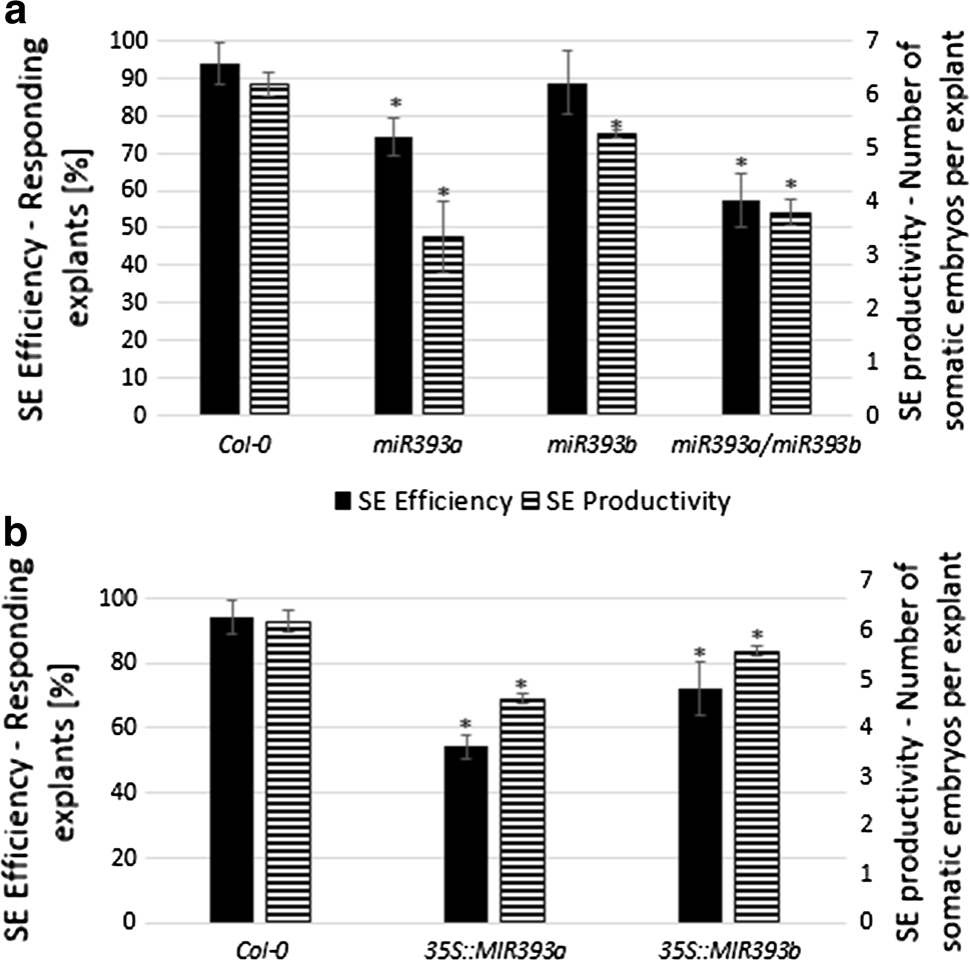
SE efficiency and SE productivity of the cultures derived from a *miR393* mutants (**a**) and the lines with an overexpression of *MIR393* genes (**b**). IZE explants were cultured on an E5 medium. *Asterisks* values significantly different from the parental Col-0 genotype (*P* < 0.05; *n* = 3 ± SD)

To further validate the involvement of *MIR393* genes in the induction of SE, transgenic lines with a contrasting activity of these genes were analysed, including T-DNA insertion mutants (*miR393a*, *miR393b*, *miR393a/miR393b*) and overexpressor lines (*35S::MIR393a*, *35S::MIR393b*). The embryogenic potential of these genotypes was examined in vitro and the derived cultures were found to be significantly impaired in their capacity for SE in comparison to Col-0 (Fig. 3). We observed that both parameters, the frequency of the embryogenic explants and the average number of somatic embryos, were significantly reduced in the cultures derived from the mutants (Fig. 3a) and the overexpressor lines (Fig. 3b). The obtained results indicated that modulation of the level of miR393 molecules affects the embryogenic capacity of explants, thus suggesting the impact of miR393 on the regulation of SE induction in Arabidopsis.

### miR393 targets

**Fig. 4:**
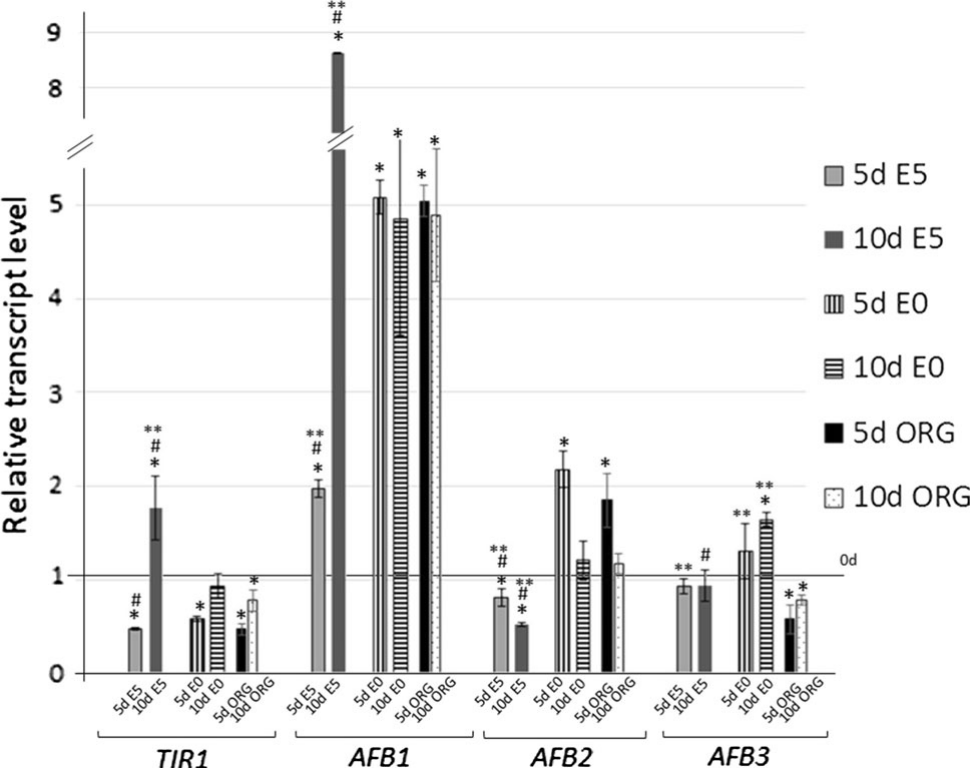
Expression of miR393 target genes (*TIR1*, *AFB1*, *AFB2* and *AFB3*) in IZE explants of Col-0 cultured on an auxin-free (E0), auxin (E5) and auxin with cytokinin (ORG) medium. Relative transcript level was normalised to the internal control (*At4g27090*) and calibrated to the 0 day of culture. *Bars* represent standard deviation. *Asterisks* values significantly different from the 0 day of culture; *hash* values significantly different from the E0 culture of the same age; *double asterisks* values significantly different from the ORG culture of the same age (*P* < 0.05; *n* = 3 ± SD)

We investigated the expression of *TIR1*, *AFB1*, *AFB2* and *AFB3* genes, which are targeted by miR393 in Arabidopsis (Navarro et al. 2006), in the IZE cultures induced on auxin (E5), auxin and cytokinin medium (ORG) and auxin-free (E0) medium using real-time qRT-PCR. The results of the analysis indicated diverse expression patterns of the target genes during SE (E5) and except for *AFB3*, the level of three other miR393-targeted transcripts were significantly modulated in the embryogenic culture (Fig. 4). A down-regulation of *TIR1* and *AFB2* at an early stage of SE induction (5 days) was observed and later on, a further reduction of *AFB2* transcripts was evident while *TIR1* expression was increased at a more advanced SE stage (10 days). In contrast to *TIR1* and *AFB2*, a gradual up-regulation of *AFB1* was observed during SE and the gene transcripts were accumulated over eight-fold at more advanced SE (10 days).

In contrast to SE, the expression profile of the auxin receptor genes in the ORG culture was similar to that observed in the control culture induced on a hormone-free (E0) medium. Thus, the significantly modulated transcript level of *TIR1/AFB1/AFB2* that was observed exclusively during SE appears to be associated with embryogenic transition and not to the auxin treatment per se.

**Fig. 5:**
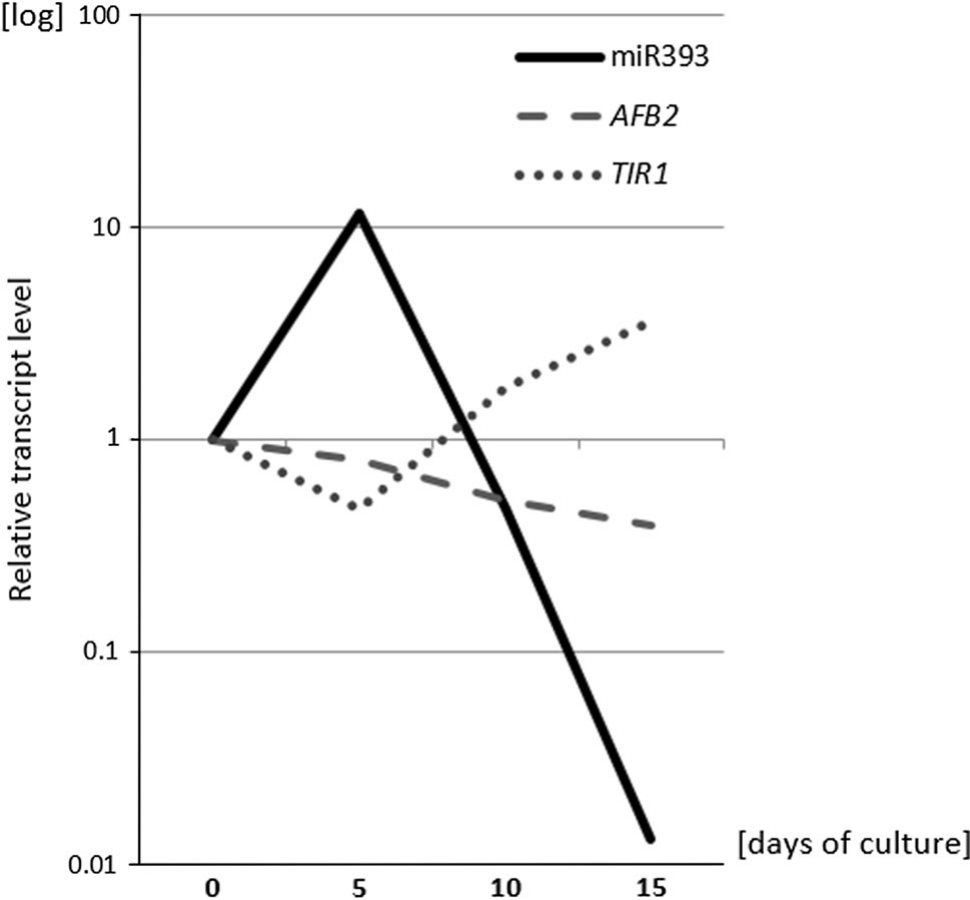
Relative amount of mature miR393 molecules and transcript level of *TIR1* and *AFB2* genes at 0, 5th, 10th and 15th day of SE culture of Col-0. Relative transcript level was normalised to the internal control (*At4g27090*) and calibrated to the 0 day of culture (*n* = 3)

A comparison of the levels of mature miR393 molecules and target transcripts in the embryogenic culture provided further support for a regulatory link between miR393 and *TIR1* and *AFB2* during SE (Fig. 5). We found that during an early stage of SE induction (5 days), the level of miR393 was opposite to *TIR1* and *AFB2*, thus implying that miR393 might down-regulate these targets during the early phase of SE induction. In a more advanced SE culture, the sharply decreasing level of miR393 was related to the accumulation of *TIR1* but not of *AFB2* transcripts.

**Fig. 6:**
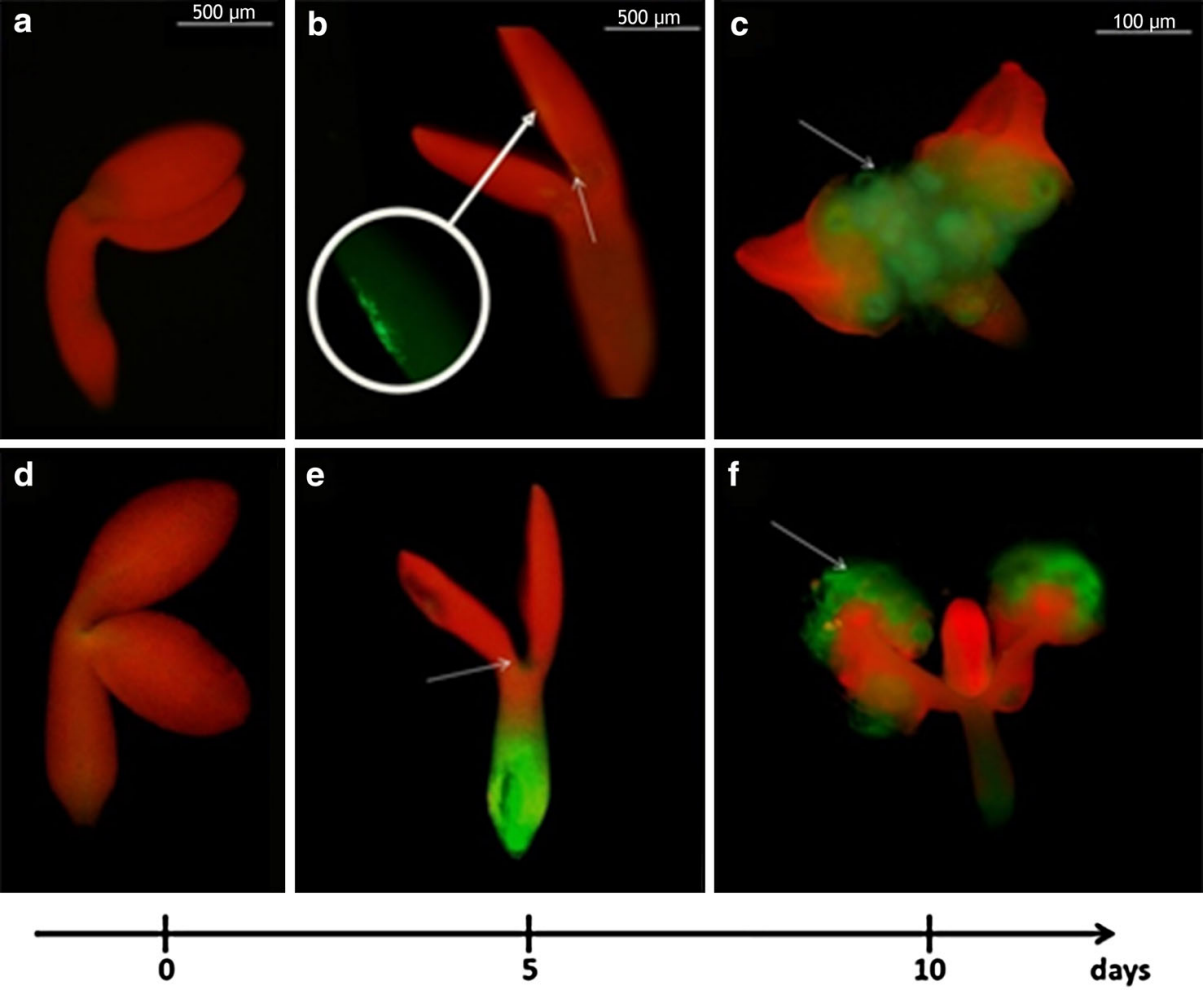
GFP-monitored expression patterns of miR393-target genes (*TIR1* and *AFB2*) in IZE explants cultured on an SE induction E5 medium. GFP signal (*green*) indicative for *TIR1* (**a–c**) and *AFB2* (**d–f**) at 0 day (**a**, **d**), 5 days (**b**, **e**) and 10 days (**c**, **f**) of SE culture. *Red* signal shows autofluorescence of chlorophyll, *white arrows* point to GFP signal localised at the explant areas involved in SE induction. *Bar* 500 µm (**a**, **b**, **d**, **e,** 0 day and 5 days), 100 µm (**c**, **f**, 10 days)

**Fig. 7:**
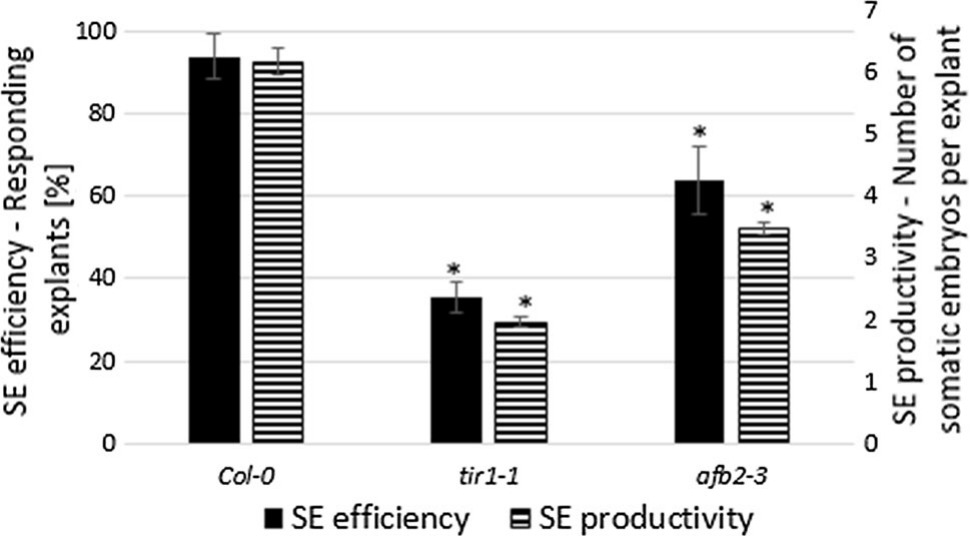
SE efficiency and SE productivity of the *tir1*-*1* and *afb2*-*3 TIR1* and *AFB2* mutant explants cultured on an E5 medium. *Asterisks* values significantly different from the parental Col-0 genotype (*P* < 0.05; *n* = 3 ± SD)

To gain insight into the spatio-temporal expression pattern of *TIR1* and *AFB2* genes in SE, the explants of the reporter lines (*TIR1pro:EGFP*, *AFB2pro:EGFP*) that were undergoing embryogenic induction on the E5 medium were inspected. No GFP signal was detected in the freshly isolated explants (0 day) but an intense signal reflecting the expression of *TIR1* and *AFB2* in the explant parts that are involved in the embryogenic transition (cotyledons, proximity of the shoot apical meristem, SAM) was observed at an early (5th day) and an advanced stage of SE (10th day) (Fig. 6). However, in contrast to *TIR1*, whose signal was distributed exclusively in the SE-associated parts of the explants, the GFP signal reflecting *AFB2* expression on the 5th day of SE was also found in the basal part of the explants, which are not involved in SE. The results of the GFP analysis infer that *TIR1* and *AFB2* transcription is associated with the explant parts that are involved in SE induction. In contrast to *TIR1* and *AFB2*, the GFP-signal of two other auxin receptor genes, *AFB1* and *AFB3*, was not observed in the explant parts that are engaged in SE (Supplementary Fig. S3). In support for *TIR1* and *AFB2* involvement in SE, *tir1*-*1* and *afb2*-*3* insertional mutants were found to be significantly impaired in the embryogenic response and both the SE efficiency and productivity of the mutant cultures were significantly reduced (Fig. 7).

**Fig. 8:**
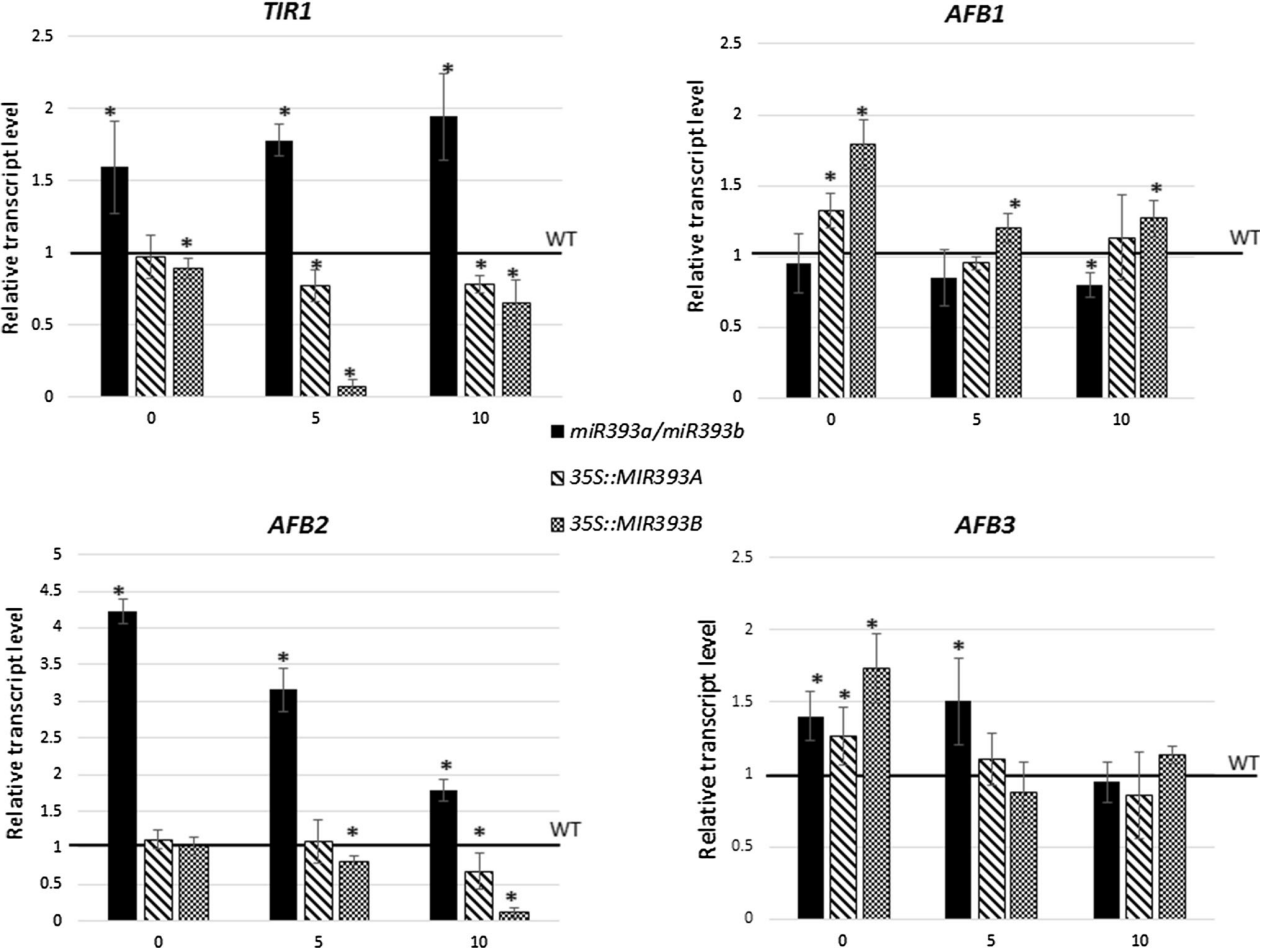
Expression of miR393 target genes (*TIR1*, *AFB1*, *AFB2* and *AFB3*) in IZE explants of *miR393a/miR393b*, *35S::MIR393a* and *MIR393b* lines. Relative transcript level was normalised to the internal control (*At4g27090*) and calibrated to the WT culture. *Bars* represent standard deviation. *Asterisks* values significantly different from the WT derived culture of the same age (*P* < 0.05; *n* = 3 ± SD)

To further verify the hypothesis that miR393 regulates SE induction by targeting *TIR1* and *AFB2* expression, the transcription of the miR393-target genes in an SE culture derived from transgenic lines with a disturbed level of miR393 was analysed (Fig. 8). The results, which show a reduction of *TIR1* and *AFB2* transcripts in the *MIR393* overexpression lines and their accumulation in the *miR393a/miR393b* mutant, suggest a regulatory relation between miR393 and *TIR1/AFB2* genes in SE. In contrast to *TIR1* and *AFB2*, two other auxin receptor genes, *AFB1* and *AFB3*, appear not to be targeted by miR393 in the embryogenic transition of somatic cells.

### miR393 affects explant sensitivity to auxin treatment

Given that miR393 impacts SE induction through the regulation of the *TIR1* and *AFB2* genes that encode auxin receptors, we hypothesised that the biological function of miR393 in the control of embryogenic transition is the modulation of a tissue’s sensitivity to exogenous auxin.

**Fig. 9:**
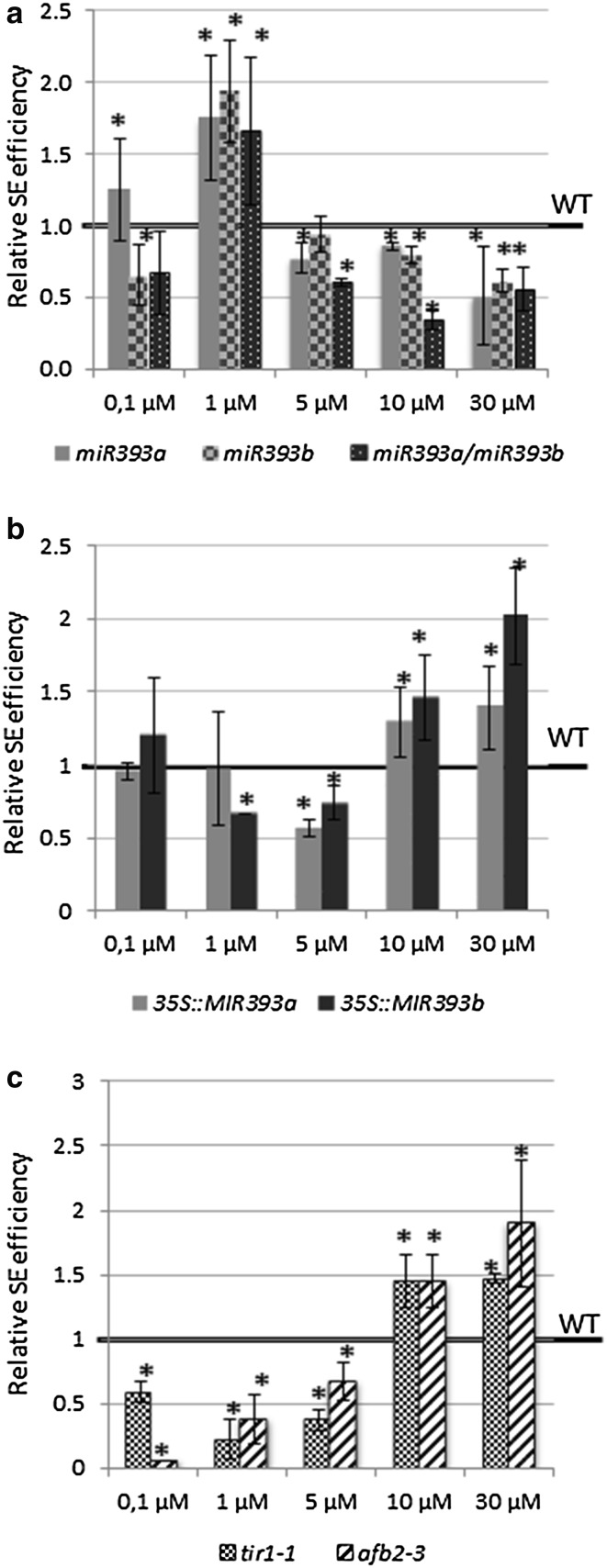
SE efficiency of the transgenic lines with a different activity of miR393 due to an insertion in *MIRNA* genes, *miR393a; miR393b*, *miR393ab* (**a**), overexpression of *MIR393* genes, *35S::MIR393a*, *35S::MIR393* (**b**) and insertion in miR393-targeted genes (**c**), *TIR1* and *AFB2*-*3* genes (*tir1*-*1*, *afb2*-*3*). IZE explants were cultured on an SE induction medium with different concentration of 2,4-D. *Asterisks* values significantly different from the parental Col-0 genotype cultured on the same medium (*P* < 0.05; *n* = 3 ± SD)

To verify this hypothesis, we analysed the embryogenic potential of explants with a disturbed accumulation of miR393 molecules and *TIR1/AFB2* transcripts in relation to different concentrations of 2,4-D added to the SE induction medium. Explant treatment with 5 µM of 2,4-D was the most effective for SE induction in Col-0 (WT) culture, which is in agreement with the optimised protocol for SE-induction in Arabidopsis (Gaj 2001) (Supplementary Fig. S2). Analysis of the cultures derived from the *miR393* insertional lines indicated that these mutants show the highest embryogenic response under treatment with 1 µM of 2,4-D, which is a concentration that is suboptimal for SE induction in Col-0 (Fig. 9a). In contrast to the *miR393* mutants, the lines overexpressing *MIR393* exhibited a significantly reduced sensitivity to auxin treatment and their explants formed somatic embryos efficiently in the presence of 30 µM of 2,4-D (Fig. 9b), which is a concentration that was over the one that was optimal for SE induction in the Col-0 culture. Like the *MIR393* overexpression lines, the *tir1*-*1* and *afb2*-*3* mutant cultures exhibited an extremely reduced sensitivity to auxin treatment and their embryogenic response was significantly enhanced on the medium with the highest 2,4-D concentration (Fig. 9c).

Collectively, these results provide evidence that miR393 controls the embryogenic potential of the somatic tissues via the modulation of auxin sensitivity.

## Discussion

The molecular mechanism that contributes to the embryogenic transition of somatic cells has recently being intensively investigated and auxin-related pathways have been emerging as playing an essential function in the regulation of SE induced in vitro in plant explants (Su et al. 2009; Bai et al. 2013; Wójcikowska et al. 2013). The reports on the central role of the transcriptional regulation of auxin metabolism/signalling in SE induction implies that microRNAs, which are frequent regulators of transcription factor gene expression, might have a substantial impact on triggering embryogenic development in the somatic cells of plants.

The results on the analysis of *dcl* mutants provided in the present study support the postulate on the engagement of miRNA in SE regulation. It was observed that the null mutation in *DCL1* (*dcl1*-*6*), but not the mutations in other *DCL* genes (*dcl2/3/4*), was found to totally eliminate the capacity of the explants for SE. Considering that DCL1, in contrast to the other three DCL proteins (DCL2-4) encoded in Arabidopsis genome, controls the processing of most of the miRNAs (Rogers and Chen 2013), the inability of the *dcl1* mutant for SE infers a key role of miRNAs in the embryogenic transition of somatic cells. Similar to the present observation on the contrasting SE phenotypes of *dcl1*-*6* and *dcl2/3/4* mutants, *dcl1* null mutants in ZE were found to be highly defective in embryo development while a triple *dcl2/3/4* mutation did not affect the plant phenotype (Bouché et al. 2006; Nodine and Bartel 2010; Willmann et al. 2011; Seefried et al. 2014). Thus, the convergent effects of *dcl1* mutations in SE and ZE infer that miRNAs that is processed by DCL1 controls the embryogenic development of generative and vegetative cells. The key regulators of embryogenic development were indicated among the miRNA-controlled genes, including *LEC2*, which has central functions in ZE and SE (Braybrook and Harada 2008; Ledwon and Gaj 2011; Willmann et al. 2011). *LEC2* was found to be under the indirect regulation of miR166, which is processed by DCL1 (Zhu et al. 2013; Jia et al. 2014). Thus, the de-regulation of the genes that regulate SE induction is assumed to account for the inability of the *dcl1* mutant for embryogenic induction.

### miR393 impacts SE induction

To gain more insights into the molecular mechanism of the miRNA-mediated regulation of SE induction, we found it to be reasonable to focus on the auxin-related regulators of gene transcription. Hence, the *MIRNA393* genes that have a substantial role in the regulation of the auxin responses by targeting auxin receptors (Chen et al. 2011b; Iglesias et al. 2014) were subjected to the analysis in the present study. Our observation that within numerous *MIRNA* genes with a significantly up-regulated transcription in SE, the *MIRNA393a* and *MIRNA393b* primary transcripts were highly accumulated (K. Szyrajew, D. Bielewicz, Z. Szweykowska-Kulińska, A. Jarmołowski and M. D. Gaj, data not shown) supports the engagement of these genes in the regulation of embryogenic transition. The contribution of both of the *MIR393* genes encoded in the Arabidopsis genome to miR393 production might be expected considering that stress- and auxin-related functions, which have been reported for *MIRNA393a* and *MIRNA393b*, respectively, are believed to control embryogenic transition in the somatic tissue of plants (Navarro et al. 2006; Chen et al. 2011b). The activity of *MIRNA393a* and *MIRNA393b* in the SE-involved parts (cotyledons and in the vicinity of the shoot apical meristem) of the explants was shown using GUS-reporter lines, which further supports the involvement of the *MIR393* genes in SE induction. The results of the GUS and the qRT-PCR monitored gene expression also indicate the high activity of the *MIR393* genes in the early (5 days) and the advanced (10 days) phase of SE induction. In contrast, a large accumulation of mature miR393 was observed exclusively at day five of SE while a drastic decrease in miR393 was found in the most advanced culture (10 days). The post-transcriptional regulation that is involved in the production of functional miRNA in plants and animals may be considered to explain this apparent discrepancy in the levels of the primary and mature miRNA (Rüegger and Großhans 2012; Zielezinski et al. 2015). Due to the complex, multi-step and spatio-temporal control of miRNA biogenesis, the primary *MIRNA* transcripts and the relevant mature miRNA frequently appear to not be related (Obernosterer et al. 2006; Thomson et al. 2006; Szweykowska-Kulinska et al. 2013). However, in spite of the complexity of the spatio-temporal control of miRNA biogenesis, a defined SE-specific level of the primary *MIR393* transcripts seems to be required for the efficient induction of SE because the de-regulation of the *MIR393* gene expression via the overexpression and down regulation of *MIR393a* and *MIR393b* resulted in a significantly defective embryogenic potential of the culture (present results).

### miR393 targets auxin receptor genes *TIR1* and *AFB2* during SE induction

The results of the present study, which is based on gene expression profiling and an analysis of the mutant/reporter lines, suggest that miR393 is likely to impact SE induction via the regulation of two of *TAAR* genes, *TIR1* and *AFB2*. Considering that expression of *TAAR* genes is not responsive to auxin (Parry et al. 2009), the significant modulation of *TIR1* and *AFB2* transcripts observed in the SE culture (present results) appears to reflect the involvement of miR393 in embryogenic transition. Both genes encode auxin receptors, which are positive regulators of auxin signalling and they are expected to play more important roles in plant development than other members of *TAAR* clade (Parry et al. 2009; Wang and Estelle 2014). *TIR1* and *AFB2* were indicated to be targeted by miR393 in root development in Arabidopsis (Parry et al. 2009) and during salt stress in Arabidopsis (Iglesias et al. 2014) and *O*. *sativa* (Xia et al. 2012). The postulate on the miR393-controlled activity of *TIR1* and *AFB2* in SE induction in Arabidopsis, down-regulation of these genes possibly via miR393 was also observed during SE in cotton, which is relevant to the present study (Yang et al. 2012, 2013).

The present results showed that in contrast to *TIR1* and *AFB2*, two other genes of the auxin receptors of the TAAR family, *AFB1* and *AFB3*, do not seem to be regulated by miR393 in SE. Even though the translational level of *AFB1* regulation by miR393 may be considered (Navarro et al. 2006), the engagement of *AFB1* in SE is of a low probability since no GFP-monitored transcription of *AFB1* was detected in the explant parts that were undergoing SE induction (Supplementary Fig. S3). Additionally, the contribution of *AFB3* to the mechanism of SE induction appears to be doubtful considering the stable expression of this gene in the SE culture and the results of the *AFB3pro::EGFP* reporter line analysis. Moreover, the involvement of *AFB3* in SE seems to be unlikely due to the fact that the gene was indicated to control the root responses to nitrate while its function in the regulation of ZE, the zygotic counterpart of SE, was not reported (Vidal et al. 2010; Si-Ammour et al. 2011).

The drastic differences that were found between the TIR1/AFBs receptors in their affinity to both AUX/IAA proteins and auxin imply that a specific combination of different F-box receptors may regulate various auxin-regulated processes including SE (Calderón Villalobos et al. 2012). Importantly for the 2,4-D-induced SE, TAAR proteins also were demonstrated to differ in their binding affinity to auxin herbicides, and, e.g., a strong affinity of AFB5 to picloram was reported (Calderón Villalobos et al. 2012). Thus, the recognition of TAAR receptors with the highest affinity to 2,4-D, which is commonly used to induce SE, would be helpful in the identification of the miR393-targeted auxin receptors that operate in SE induction.

### miR393-mediated regulation of receptor genes *TIR1* and *AFB2* controls tissue sensitivity to auxin

miR393 was recently suggested to target the auxin receptors TIR1 and AFB2 during SE induction (Yang et al. 2013). Thus, we hypothesised that miR393 contributes to the SE induction mechanism via the modulation of tissue sensitivity to auxin. In support of this assumption, the embryogenic response of explant tissue was found to be related to the level of *MIRNA* gene expression and the concentration of 2,4-D that was used in the SE induction medium. Accordingly, the mutations in *MIRNA393* genes resulted in a hypersensitivity to auxin while the overexpression of *MIR393* transcripts led to a contrasting phenotype. Consistent with these results, the explants isolated from *tir1*-*1* and *afb2*-*3* mutants were indicated to display a hyposensitivity to auxin treatment. A similar relation between the overexpression of *MIR393* and tissue sensitivity to auxin was also observed in *Oryza sativa* in planta and a high miR393 activity was suggested as causing the decreased sensitivity of seedlings to 2,4-D that resulted in the dramatic inhibition of the primary root elongation (Bian et al. 2012).

Conclusively, we assumed that miR393 might influence the embryogenic potential of explant cells via the auxin signalling pathway and the modification of a cell’s sensitivity to auxin.

### Stress-related functions of miR393 in SE

Recently, it has become apparent that the auxin responses that accompany SE induction are closely linked to the stress responses that are activated during the embryogenic transition of somatic cells of plants (Fehér 2015). In support of the stress-related mechanism of SE induction, the enhanced production of reactive oxygen species (ROS) that was observed after 2,4-D treatment and the recently uncovered ROS-mediated modifications of the cytoskeleton structure and peroxisome dynamics, might be considered to contribute to SE induction (Pasternak et al. 2002; Ötvös et al. 2005; Rodríguez-Serrano et al. 2014). Relevant to the notion about the role of stress-responses in SE induction, several reports have documented the involvement of miR393 in the responses of plants to biotic and abiotic stresses (Navarro et al. 2006; Chen et al. 2015). A recent report on the *mir393* mutants has provided a clue for a possible role of miR393 in the control of the stress-related pathway that is activated during SE induction (Iglesias et al. 2014). Mutants with a reduced activity of miR393 were found to display an increased production of ROS, which was coupled with the repressed metabolism of the antioxidants, while the *tir1afb2* mutant displayed the opposite phenotype (Iglesias et al. 2014). In conclusion, the present results on the miR393-mediated control of SE induction provide convincing evidence that the function of miR393 in the embryogenic transition of somatic cells seems to be related to the integration of the environmental cues and the maintenance of a proper homeostasis of auxin signalling as was proposed for in planta development (Windels and Vazquez 2011; Windels et al. 2014).

Further analyses are required to define other components of the miR393-regulated network that underlie the developmental plasticity of plant cells that is released under in vitro conditions. The complex feedback regulations between the miR393 and TAAR proteins and the tissue-specific functions of the auxin signalling components might be expected during SE induction as was indicated in the developmental processes in planta (Si-Ammour et al. 2011; Chen et al. 2015; Wang and Estelle 2014).

#### *Author contribution statement*

MDG and AMW conceived and designed research. AMW conducted the experiments. AMW and MDG analysed the data and wrote the manuscript. Both of the authors read and approved the manuscript.

## Electronic supplementary material

Below is the link to the electronic supplementary material.

Supplementary material 1 (JPEG 1603 kb) Suppl. Fig. S1 Expression of pri-miR393a and pri-miR393b in IZE explants of Col-0 culture. Relative transcript level was calibrated to the 0d of SE culture. *Bars* represent standard deviation. *Asterisks* values significantly different from the 0d (*P* < 0.05; *n* = 3 ± SD)

Supplementary material 2 (JPEG 987 kb) Suppl. Fig. S2 SE efficiency and SE productivity of the IZE explants of Col-0 cultured on an SE induction medium with different concentrations of 2,4-D (*n* = 3)

Supplementary material 3 (JPEG 267 kb) Suppl. Fig. S3 GFP-monitored expression patterns of miR393-target genes (*AFB1* and *AFB3*) in IZE explants cultured on an SE induction E5 medium. GFP signal (green) indicative for *AFB1* (**a**-**c**) and *AFB3* (**d**-**f**) at 0 d (**a**, **d**); 5 d (**b**, **e**) and 10 d (**c**, **f**) of SE culture. *Red signal* shows autofluorescence of chlorophyll, *white arrows* point to GFP signal localised at the explant areas involved in SE induction. *Bar* = 500 µm (**a**,**b**,**d**,**e**, 0d and 5), 100 µm (**c**, **f**, 10d)

Supplementary material 4 (XLSX 9 kb) Table 1 Characterisation of the transgenic genotypes used in the study

Supplementary material 5 (XLSX 9 kb) Table 2 Primers used for qRT-PCR analysis

Supplementary material 6 (XLSX 9 kb) Table 3 Primers used for miRNA detection

## Abbreviations

*AFB*: *Auxin F*-*box protein*
2,4-D: 2,4-Dichlorophenoxyacetic acid
E5: Embryogenesis induction medium with auxin
E0: Auxin-free induction medium
ORG: Organogenesis
DCL: Dicer-like
*MIR*: *MICRO RNA* gene
IZE: Immature zygotic embryo
SE: Somatic embryogenesis
*TIR1*: *Transport inhibitor1*
ZE: Zygotic embryogenesis
