# Supplementary figures and images for: miR393 contributes to the embryogenic transition induced in vitro in Arabidopsis via the modification of the tissue sensitivity to auxin treatment
Source: Planta. 2016 Apr 4;244:231–43. doi: 10.1007/s00425-016-2505-7 (PMC4903112; doi:10.1007/s00425-016-2505-7)

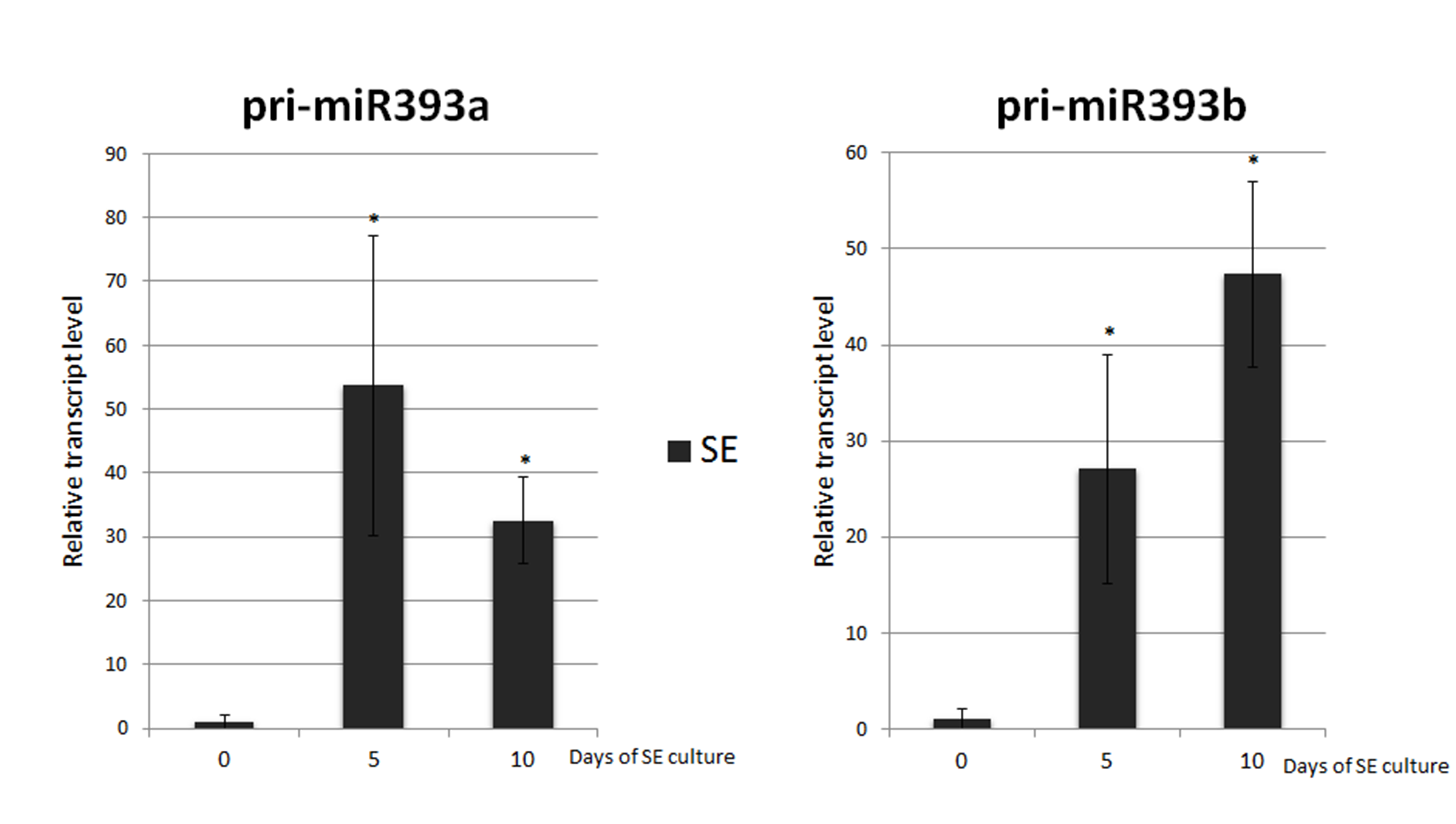

Supplement: Supplementary file 1 — Supplementary material 1 (JPEG 1603 kb) Suppl. Fig. S1 Expression of pri-miR393a and pri-miR393b in IZE explants of Col-0 culture. Relative transcript level was calibrated to the 0d of SE culture. Bars represent standard deviation. Asterisks values significantly different from the 0d (P < 0.05; n = 3 ± SD) [file 425_2016_2505_MOESM1_ESM.jpg]

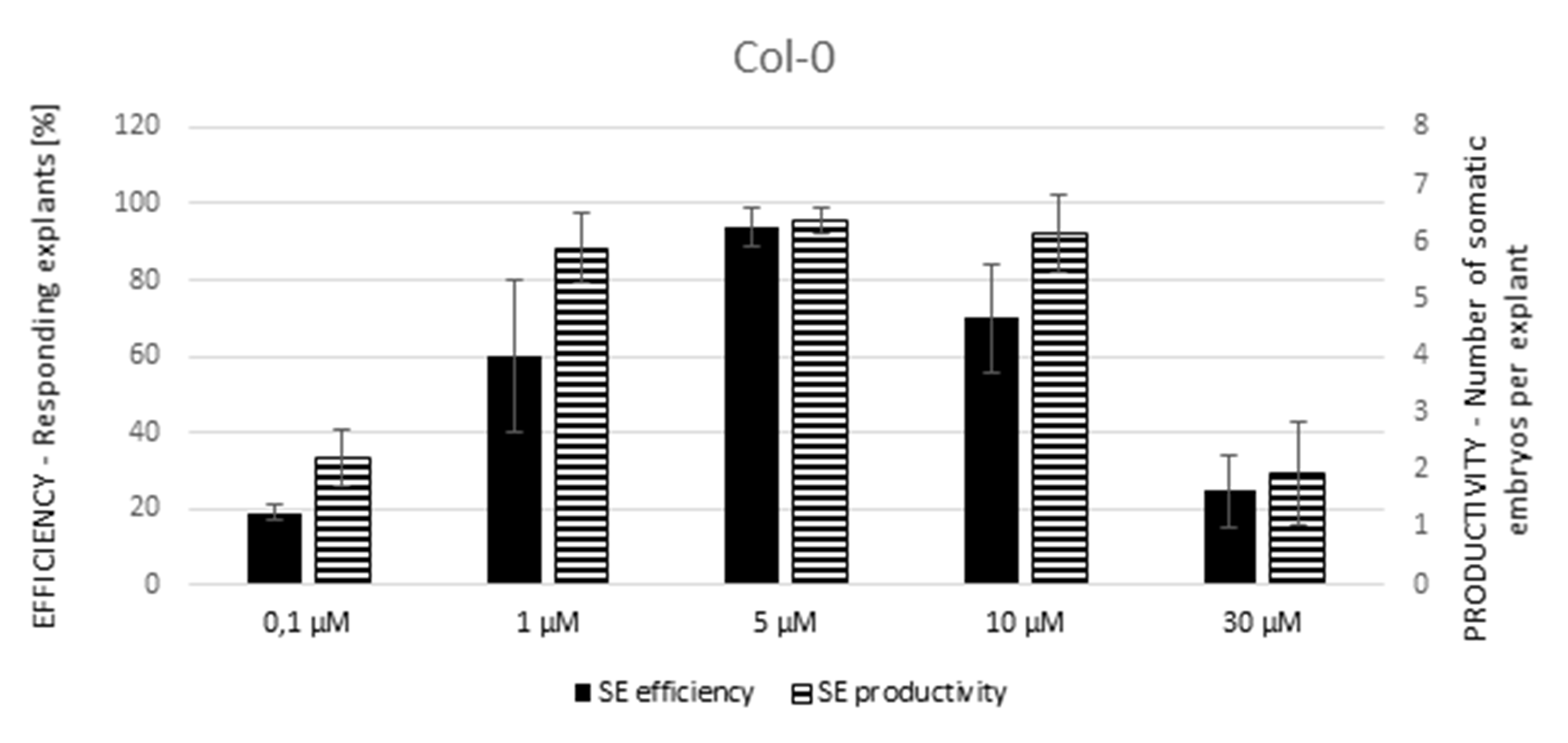

Supplement: Supplementary file 2 — Supplementary material 2 (JPEG 987 kb) Suppl. Fig. S2 SE efficiency and SE productivity of the IZE explants of Col-0 cultured on an SE induction medium with different concentrations of 2,4-D (n = 3) [file 425_2016_2505_MOESM2_ESM.jpg]

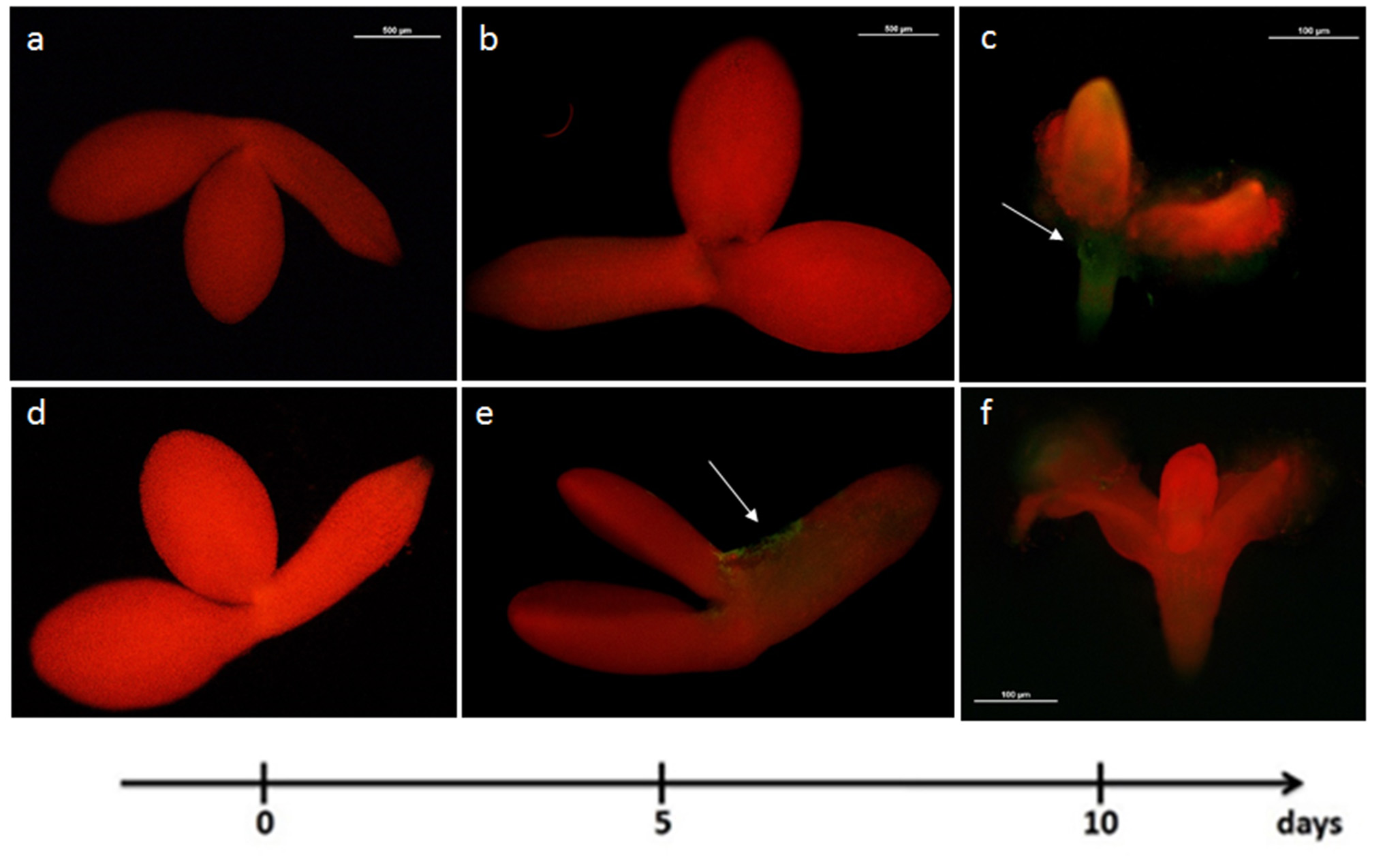

Supplement: Supplementary file 3 — Supplementary material 3 (JPEG 267 kb) Suppl. Fig. S3 GFP-monitored expression patterns of miR393-target genes (AFB1 and AFB3) in IZE explants cultured on an SE induction E5 medium. GFP signal (green) indicative for AFB1 (a-c) and AFB3 (d-f) at 0 d (a, d); 5 d (b, e) and 10 d (c, f) of SE culture. Red signal shows autofluorescence of chlorophyll, white arrows point to GFP signal localised at the explant areas involved in SE induction. Bar = 500 µm (a,b,d,e, 0d and 5), 100 µm (c, f, 10d) [file 425_2016_2505_MOESM3_ESM.jpg]
